# Supplementary material for: Novel Microwave Synthesis of Copper Oxide Nanoparticles and Appraisal of the Antibacterial Application
Source: Micromachines (Basel). 2023 Feb 15;14(2):456. doi: 10.3390/mi14020456 (PMC9960782; doi:10.3390/mi14020456)
Supplement: Supplementary file 1 [file micromachines-14-00456-s001.zip › micromachines-2178986-supplementary.pdf]

Novel microwave synthesis of antibacterial copper oxide nanoparticles in the presence of apple peel extract

Rajaram Rajamohan[a]* Chaitany Jayprakash Raorane[a], Seong-Cheol Kim[a]*, Sekar Ashokkumar[b], Yong Rok Lee[a]*

[a]School of Chemical Engineering, Yeungnam University, Gyeongsan 38541, Republic of Korea.

[b] Plasma Bioscience Research center, Kwangwoon University, Seoul 01897, Republic of Korea.

*Correspondence: rajmohanau@yu.ac.kr **(R.R);** sckim07@ynu.ac.kr **(S.C.K);** yrlee@yu.ac.kr **Y.R.L)**

2. Materials and Methods

Materials

Sigma Aldrich Company, South Korea, provides copper nitrate (molecular formula: $CuCl_2.2H_2O$, Purity: >99%) for the synthesis of NPs without further purification. $CuCl_2.2H_2O$ was readily soluble in distilled water. After thoroughly washing the glassware with distilled water, they were dried in the oven for 30.0 minutes to avoid contamination in the glassware by the deposition of impurities.

Preparation of Apple peel extract (AP extract)

A Red Delicious apple peeler was used to peel a kilogram of fresh apples. A chilled Waring blender was used to homogenize apple peels (100.0 mg, 10.3% of the fresh apple weight) for 5 minutes with chilled 80.0 % acetone (1:2, w/v). For a further 3 minutes, samples were homogenized using a Polytron homogenizer. A vacuum at 45.0 °C was used to evaporate approximately 90.0 % of the filtrate. Following the first extraction step, the residue (100.0 mg, 16.2 % apple peels) was resuspended in 500.0 mL water, extracted thrice with ethyl acetate, and then extracted with water-saturated n-butanol.

Preparation of CuO NPs

The preparation of NPs from the same precursor of $CuCl_2$. $2H_2O$ under microwave irradiation at three different temperatures. Three sets of AP extract (10.0 mL) and $CuCl_2.2H_2O$ (3.146 g/50.0 mL water) were taken separately with the required amounts. A scheme for synthesizing NPs was shown in **Scheme S1**. An extract of AP was typically added directly to aqueous solutions of $CuCl_2.2H_2O$ at 0.003 mol/L with constant stirring for ten minutes at 60.0 °C. A pale blue color developed in the solution after ten minutes. In a microwave oven (Panasonic N-ST342), the mixture was irradiated for 300 seconds at 90.0, 100.0, and also 110.0 °C under an atmosphere of high N_2 purity. No color change in the mixture which has irradiated at 90.0 °C. The other two mixtures turned the color from pale blue turned to light brown in less than a minute. A 15-minute centrifuge with three 5-minute intervals was used to separate the CuO NPs from the solvent medium, followed by several washes with ethanol and deionized water. Centrifugation causes CuO NPs to sediment after washing, followed by a sonicator in water for one minute. A refrigerator was used to store the CuO NPs obtained through this process after they were dried for one day at 400.0 °C. The final NPs denoted as CC 100, and CC 110. It was highly probable that CuO nanoparticles will form NPs when exposed to air. AP extract was an efficient reducing and stabilizing agent in the formation of NPs.

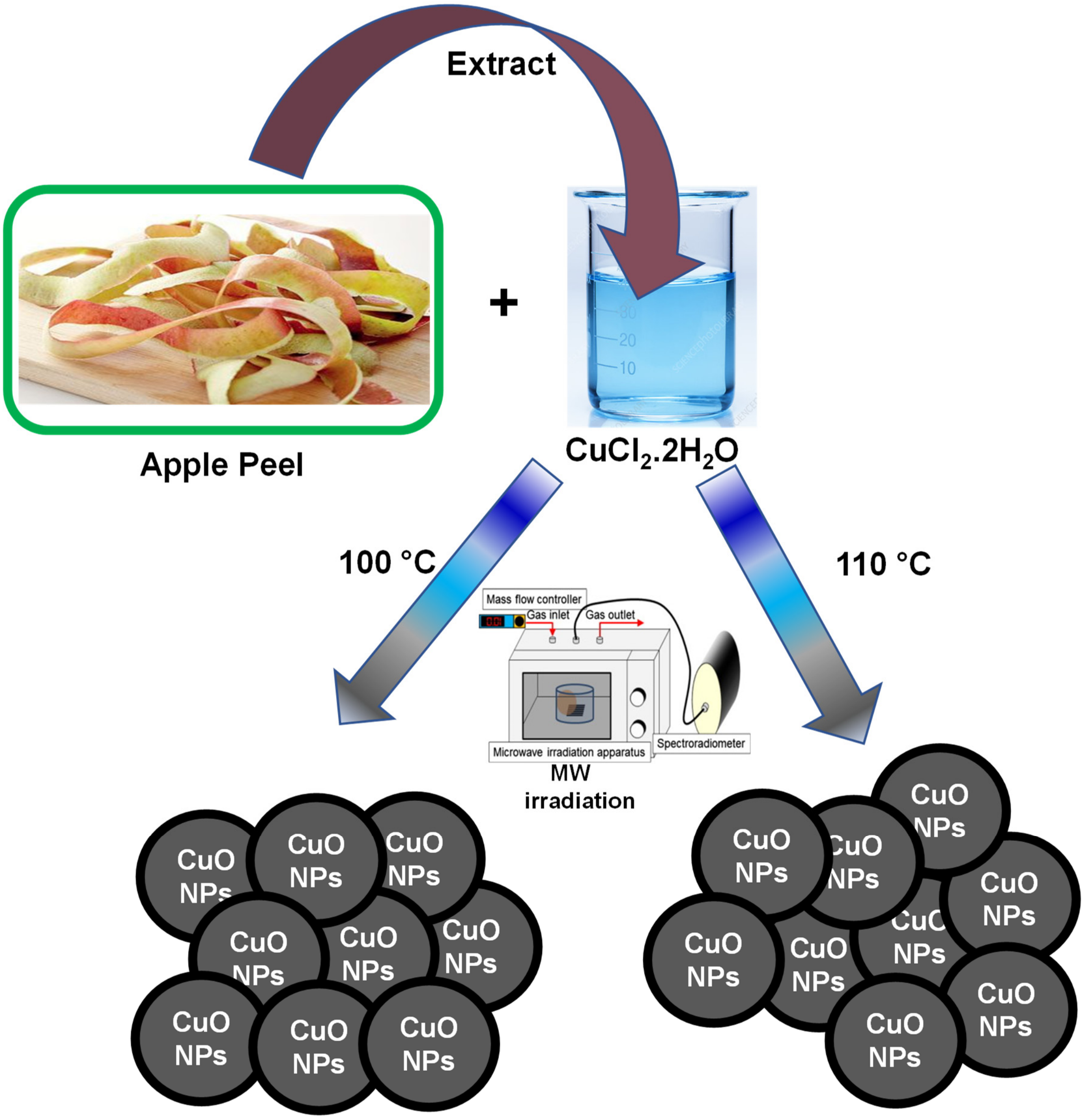

Scheme S1. Synthesis of CuO NPs

MDA MB 231 cell culture

The MDA MB 231 (human breast cancer cell) cells are cultured in a T-75 flask containing Dulbecco's Modified Eagle Medium (DMEM) supplemented with 10% fetal bovine serum and 1% antibiotic (streptomycin & penicillin). The MDA MB-231 inoculated plates are incubated at 37 °C in 5% CO_2 for up to 2 days, and the cells reached ~80% confluence to start the cell viability experiment. To maintain the cells in the exponential growth phase, they are passaged every three days at a 1:3 ratio.

Cytotoxicity (*in-vitro* cell viability)

The *in-vitro* cell viability of the Cu_2O NPs is investigated Alamar blue assay. Briefly, MDA MB 231 cells are seeded into 96 well plates at a density of cells (2×10^4 cells/well) when cells reached 80% confluence (2 days). Then, the old-growth medium is replaced with 200 µl of fresh medium containing different concentrations of derivatives (2.0, 1.8, 1.6, 1.4, 1.2, 1.0, 0.8, 0.6, 0.4, and 0.2 mg/ml) is added into each well and incubated for 1, 2 and 3 days. Based on diverse instance periods, the plate is washed well and a further10% of Alamar blue is added, incubated at 37 °C with 5% CO_2 for up to 4 hours, and the plate is covered with protection from direct attack of sunlight. The plate read at excitation and emission wavelength of 540.0 and 600.0 nm (absorbance value) using a microplate reader Synergy HT spectrophotometer (BioTek, Winooski, VT, USA).

Apoptosis study

MDA MB 231 cells are seeded into 6 well plates at a density of cells (2×10^5 cells/well), and the cells reached 80 to 90% confluence. The culture medium is removed and replaced immediately with a fresh medium containing 1mg/ml, and then blank NPs are incubated for about 4 hrs. Floating and adherent cells are collected, centrifuged at 1500 rpm for 5 min, and the pellets are washed twice with cold PBS solution and resuspended. The cells are added 100µl of 1X binding buffer, 5.0 µl of Annexin V-FITC, and propidium iodide (PI) respectively, and incubated for 15 minutes at room temperature. At the end, cell apoptosis is measured with flow cytometry (BD FACSVerse, BD Biosciences, NJ, USA).

Instruments Used

Various techniques were used to analyze the NPs, including diffusion reflectance spectroscopy, FT-IR spectroscopy, Raman spectroscopy, FE-SEM with EDX spectroscopy, HR-TEM, XRD, XPS, TGDTA, and BET surface area analysis. DRS is measured using an OPTIZEN 3220 UV spectrophotometer within the wavelength ranges of 200.0 - 800.0 nm. FT-

IR spectra are recorded with the Perkin Elmer Spectrum Two in transmittance mode within the ranges of 400.0 - 4000.0 cm^{-1}. 16 scans for the measurement at a resolution of 8 cm^{-1}. Raman spectral measurements were captured on the XploRA Micro-Raman spectrophotometer (Horiba) within the spectral shift ranges of 100.0 - 1500.0 cm^{-1}. At a 10.0 kV accelerating voltage, FE-SEM, and EDX spectral analysis was carried out with the Hitachi S-4800. HR-TEM images are captured using an FEI-Tecnai TF-20 with a 120.0 kV operating accelerating voltage. Powder XRD measurements were performed on a PANalytical X'Pert3 MRD diffractometer at 40.0 kV and 30 mA with monochromatized Cu K radiation (λ =1.54 Å). The 2θ range is 10° - 80° at a scan rate of 5° min^{-1} and a wavelength of 1.5405 Å. K-Alpha is used to generate XPS spectra (Thermo Scientific). The CasaXPS software S3 is used to deconvolve the high-resolution XPS spectra. The thermal behavior of the NPs is performed using TA instruments, and the curves are of individual elements analyzed using the Universal V4.5A Program. The weight of each sample is around 5.5 mg and the temperature range for the measurement is about 35.0 - 700.0 °C with increments of 10 °C/minute. All of the above instrument services were utilized at the core research support center (CRSC) for natural products and medical materials at Yeungnam University, South Korea.

A probable mechanism for the formation of CuO NPs

Scheme S2 provides an overview of the proposed mechanism for making CuO NPs. As part of the AP extract-assisted CuO NPs biosynthesis process, there were usually three steps including the activation, growth, and termination phases**.** In the synthetic process, AP extract may be both a reducing and stabilizing agent. In an initial step, a $CuCl_2.2H_2O$ salt precursor would be dissolved in distilled water and activated by removing its cations. Copper ions and AP extract undergo an oxidation-reduction reaction during mixing and followed by magnetic stirring at 60.0 °C. Copper cations, for instance, were reduced from a 2+ oxidation state into a metallic form. The nucleation process (Cu^0) involves the direct conversion of electron-rich

natural constituents, such as biomolecules with hydroxyl groups, directly into CuO NPs due to the superior chemical reactivity of raw copper metal surfaces. FT-IR spectra of CuO NPs clearly show a broad peak of -OH from AP extract becomes narrower, suggesting that the alcoholic group effectively participates in the bio-reduction process. The Cu^0 was gradually combined into CuO NPs during the growth phase by the oxidation process**.** Finally, CuO NPs were stabilized during the termination step. A protective shield of linear and branched natural biomacromolecules derived from apple peel extract surrounds the nucleated NPs, preventing them from growing. Furthermore, these biological macromolecules maintain the separation of capped NPs via steric forces**.** Likewise, food waste contains various bioactive compounds that reduce metal ions and stabilize metallic NPs**.**

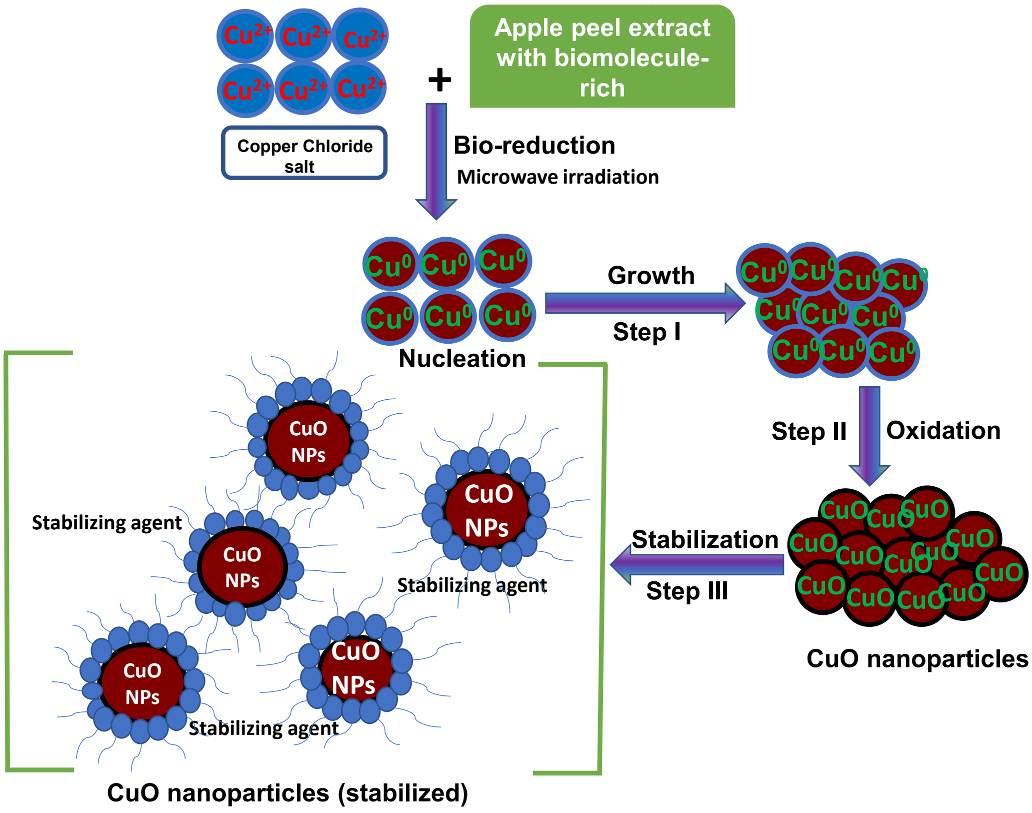

Scheme S2. Schematic pathway for the bio-reduction of CuO NPs with the use of AP extract.

In-vitro **cytotoxicity**

The synthesized CuO NPs are tested for cytotoxicity, and an assay plays a vital role in the cell lines. The study is used to determine cell survival, death, and metabolism. *In vitro* cell viability of the NPs is performed by Alamar blue assay using the MDA MB 231 cell line and the data are expressed as mean ± SD (n = 3). The obtained results are shown in **Figure S1A**. All the concentrations cause slight on the cell lines (0.125 to 1.0 mg/ml). The fluorescence microscopic images (Bright field) shows that there are no morphological changes in the control sample (**Figure S1A**) and also the NPs on the treated MDA MB 231 cell lines (**Figure S1B and C**).

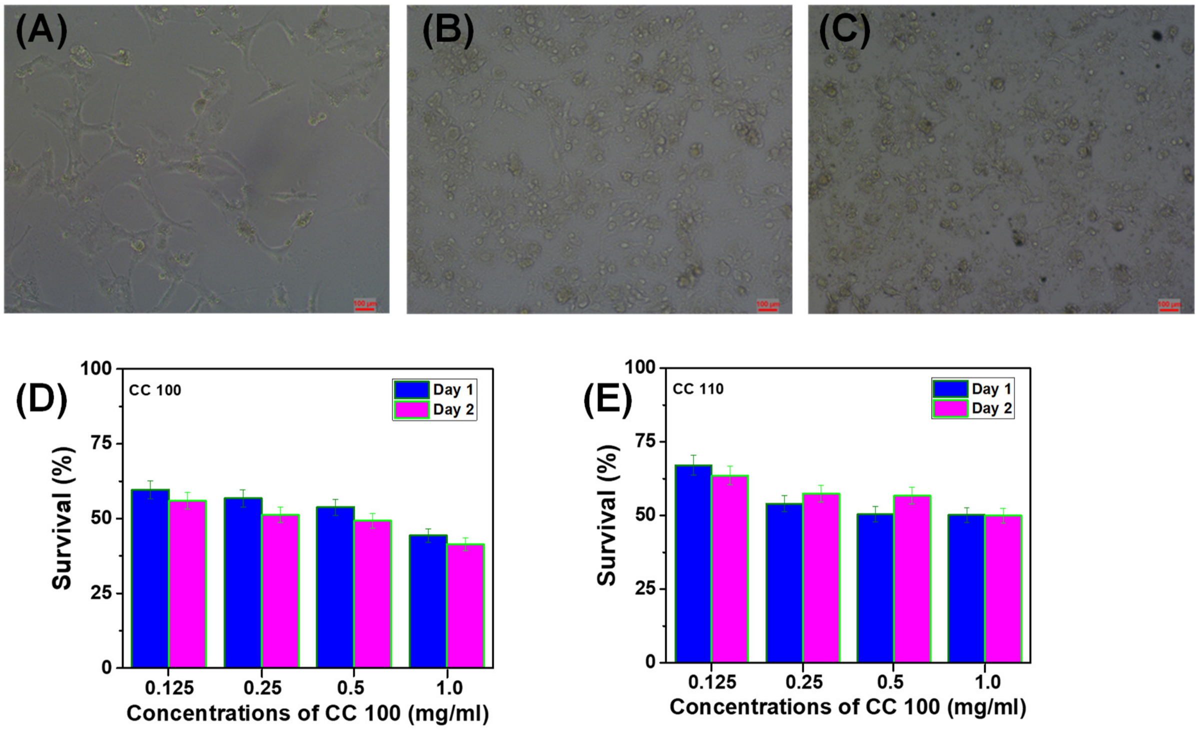

Figure S1. Fluorescence microscopic (Bright field: 10× magnification and scale bare 100µm) image of control **(A)**, Fluorescence microscopic (Bright field: 10× magnification and scale bare 100µm) image of CC 100 **(B)**, Fluorescence microscopic (Bright field: 10× magnification and scale bare 100µm) image of CC 110 **(C)**, The cytotoxicity of NPs on MDA MB 231 cell line

with CC 100 **(D)**, and the cytotoxicity of NPs on MDA MB 231 cell line with CC 110 **(E)**.
